# Examination of intrapersonal and affective characteristics associated with everyday function among older adults with subjective cognitive decline

**DOI:** 10.1002/alz.090642

**Published:** 2025-01-03

**Authors:** Sarah Tomaszewski Farias

**Affiliations:** ^1^ University of California, Davis School of Medicine, Sacramento, CA USA

## Abstract

**Background:**

The purpose of this study was to evaluate a variety of intrapersonal factors that may also be associated with everyday functional outcomes in older adults with subjective cognitive decline (SCD).

**Participants and Methods:**

Participants included 127 older adults with SCD (age *M* = 73.1, *SD* = 4.9; education *M =* 15.4, *SD* = 2.2; 80% female, 89% White) who were enrolled in an NIH funded randomized controlled trial teaching memory support strategies and healthy lifestyles (R01AG066748). This study utilized baseline data from the trial, prior to the start of the intervention. Everyday function was measured across a variety of instrumental activities of daily living (IADLs). Key independent variables of interest included self‐rated positive and negative affect, life satisfaction, resiliency, life purpose, health literacy, and perceived competence. Linear regression was used to assess associations between key independent variables and each outcome. Model building began with simple models including one key independent variable in addition to demographics (age, education, gender). A final model was then built including all variables that met a trend level association in those models (p<.15). Correlations between variables were assessed for collinearity. All analyses were conducted in R and a p‐value less than 0.05 was considered statistically significant.

**Results:**

When examining predictors of IADLs, positive affect (.001), negative affect (p = .05), resiliency (p = .1) and life purpose (p = .03) were all identified in individual models as having a potential association with IADLs after controlling for demographics. When all four variables were included in a final model, along with demographics, positive affect (p = .03) remained significantly associated with IADLs. Specifically, higher positive affect was associated with greater independence in IADLs.

**Conclusions:**

In this study we show that positive affect is an important predictor of better self‐reported IADL functioning in a group of older adults at risk for cognitive and functional decline. Positive affect has previously been associated with a host of health outcomes, but to our knowledge this is the first finding showing it may bolster everyday functioning and reduce disability. Findings may have important implications for interventions that focus on improved well‐being as a way to bolster everyday functioning among older adults with SCD.

